# 

**Published:** 1892-03

**Authors:** 


					﻿ESTABLISHED 1865.
SUBSCRIPTION, S2.0C

EDITED BY
H. v: M. MILLER, MJD., LL. D.
VIRGIL O. HARDON, M. D.
F. W. McRAE, M. D.
L.	P. KENNEDY; A.~MTm. D.
M.	B. HUTCHINS, M. D.
L. B. GRANDY, M. D.
Published by JAS. P. HARRISON & CO., Atlanta, Ga.
Entered at the Post-Office at Atlanta, Ga., aa second-class matter.
Atlanta’Ga’’March’ i892- No. i.
				

